# Supplementary material for: Risk of rapid evolutionary escape from biomedical interventions targeting SARS-CoV-2 spike protein
Source: PLoS One. 2021 Apr 28;16(4):e0250780. doi: 10.1371/journal.pone.0250780 (PMC8081162; doi:10.1371/journal.pone.0250780)
Supplement: S1 Fig — Glycosylated residues are marked in blue, while the remainder of residues are colored by the number of epitopes that contain the residue (red color bar). (PDF) [file pone.0250780.s001.pdf]

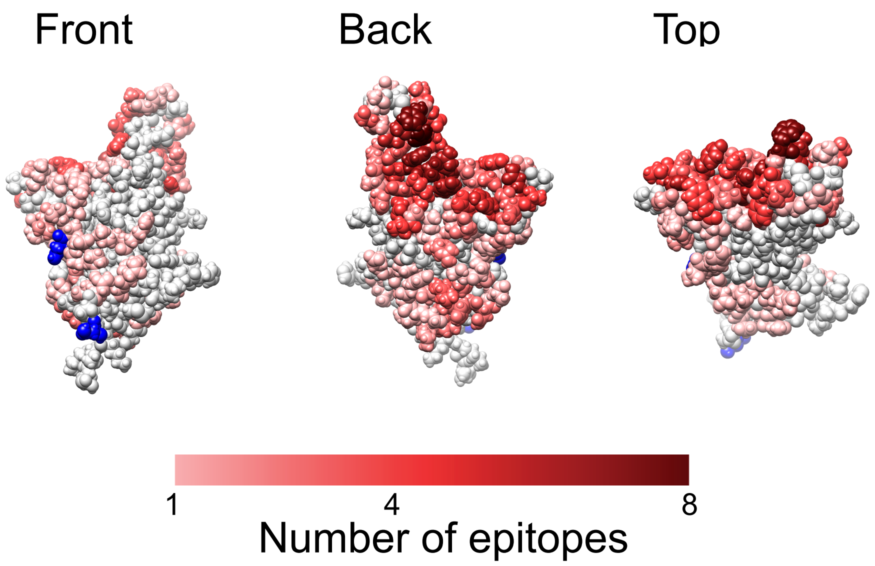

Figure S1. Glycosylation in SARS-CoV-2 spike protein RBD. Glycosylated residues are marked in blue, while the remainder of residues are colored by the number of epitopes that contain the residue (red color bar).
