# Supplementary material for: Risk of rapid evolutionary escape from biomedical interventions targeting SARS-CoV-2 spike protein
Source: PLoS One. 2021 Apr 28;16(4):e0250780. doi: 10.1371/journal.pone.0250780 (PMC8081162; doi:10.1371/journal.pone.0250780)
Supplement: S2 Fig — (PDF) [file pone.0250780.s002.pdf]

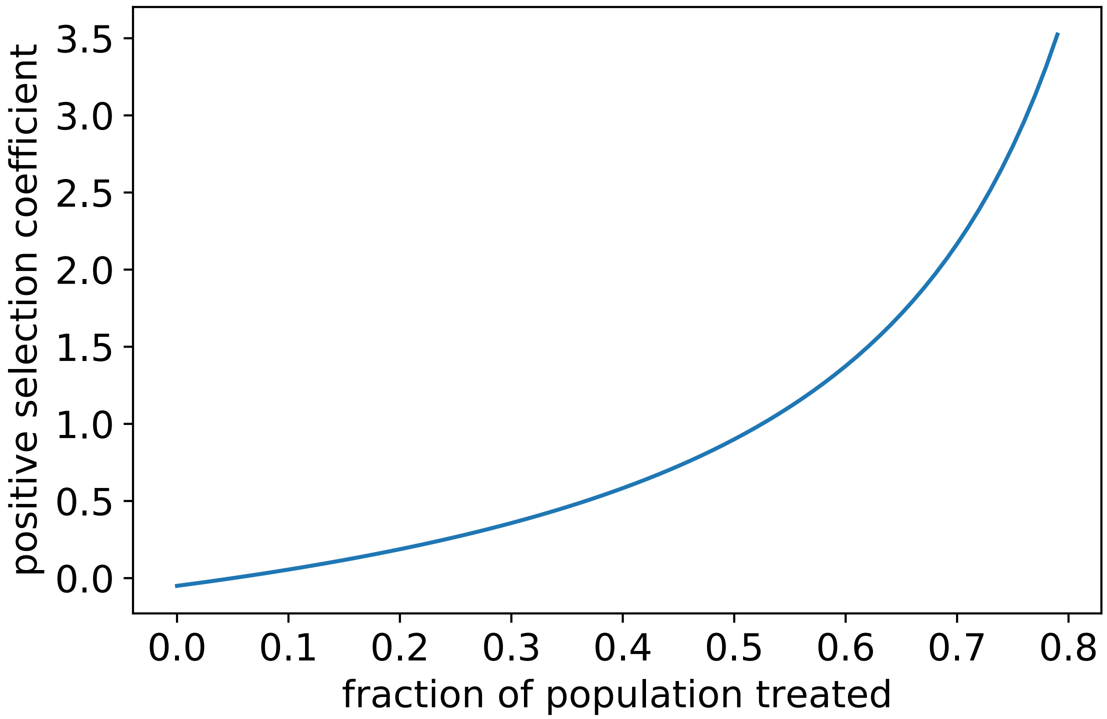

Figure S2. Relationship between the fraction of the population that receive a prophylactic that is completely effective in preventing infection from wild-type virus and the strength of selection for an escape mutant.
