# Supplementary material for: Risk of rapid evolutionary escape from biomedical interventions targeting SARS-CoV-2 spike protein
Source: PLoS One. 2021 Apr 28;16(4):e0250780. doi: 10.1371/journal.pone.0250780 (PMC8081162; doi:10.1371/journal.pone.0250780)
Supplement: S3 Fig — Time required to establish a resistant viral single (A), double (B), triple (C), or quadruple (D) mutant with different fitness costs for intermediate mutants. In our model, viral variants with some, but not all, mutations required for resistance to an antibody intervention have a fitness cost (ranging from 1–9% less infectious). Increasing the fitness cost of these intermediates prolongs the time required for a resistant variant with a specific combination of 2–4 mutations (B-D) to establish in the population. (PDF) [file pone.0250780.s003.pdf]

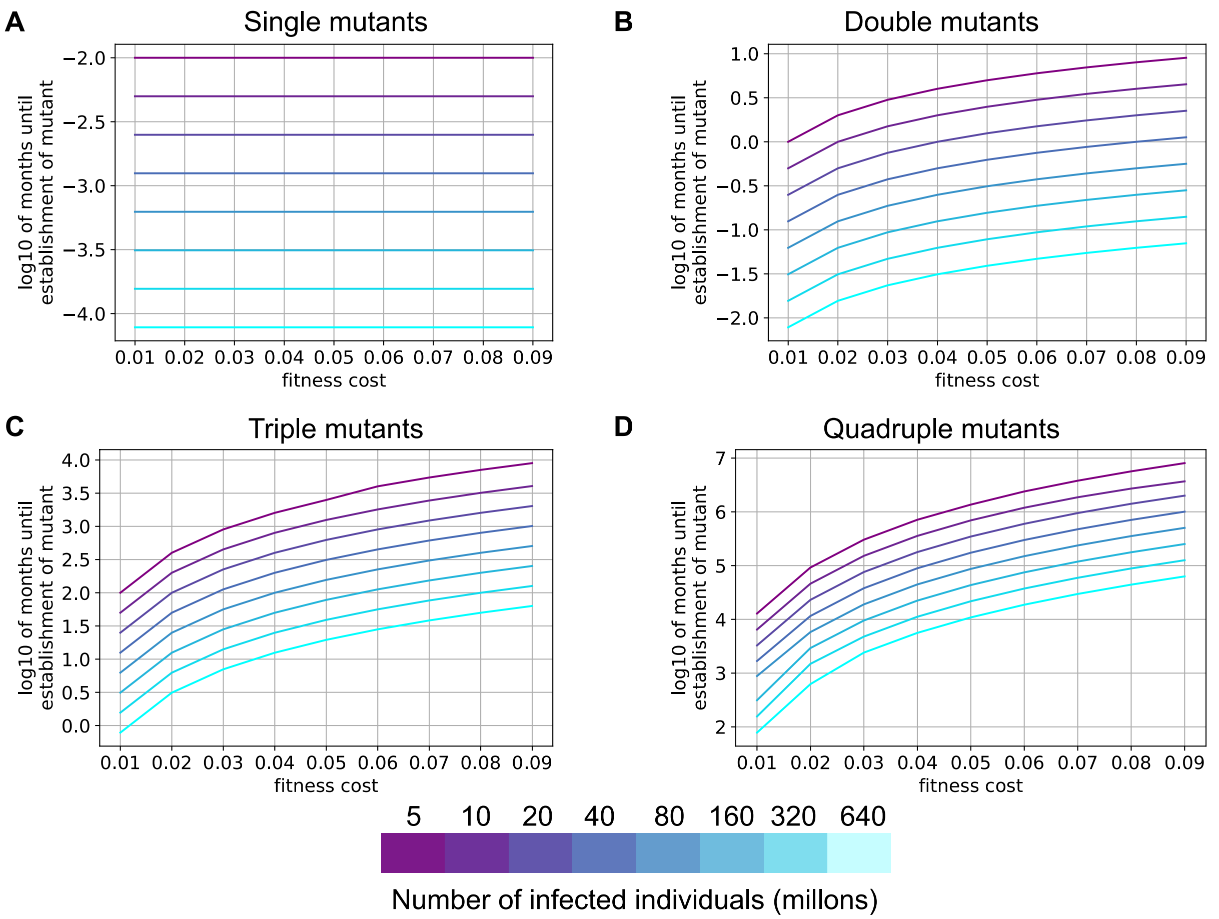

Figure S3. Time required to establish a resistant viral single (**A**), double (**B**), triple (**C**), or quadruple (**D**) mutant with different fitness costs for intermediate mutants. In our model, viral variants with some, but not all, mutations required for resistance to an antibody intervention have a fitness cost (ranging from 1-9% less infectious). Increasing the fitness cost of these intermediates prolongs the time required for a resistant variant with a specific combination of 2-4 mutations (**B-D**) to establish in the population.
