# Supplementary material for: Risk of rapid evolutionary escape from biomedical interventions targeting SARS-CoV-2 spike protein
Source: PLoS One. 2021 Apr 28;16(4):e0250780. doi: 10.1371/journal.pone.0250780 (PMC8081162; doi:10.1371/journal.pone.0250780)
Supplement: S1 Table — (PDF) [file pone.0250780.s004.pdf]

Table S1. Evolutionary rates of pathogenic RNA viruses

Virus	Type	Evolutionary rate (10^{-3} sub/site/year)	Reference
HIV	Retrovirus	2.02 – 16.8	(*1*)
Poliovirus	+ssRNA	10.3	(*2*)
Influenza A	-ssRNA	1.43 - 1.16	(*3*)
SARS-CoV	+ssRNA	7.8	(*4*)
SARS-CoV-2	+ssRNA	0.8 - 6.58	(*5*)
Rotavirus	dsRNA	0.73	(*6*)
Hepatitis C virus	+ssRNA	0.48 – 0.91	(*7*)
MERS-CoV	+ssRNA	0.24	(*4*)

1. I. M. Berry, R. Ribeiro, M. Kothari, G. Athreya, M. Daniels, H. Y. Lee, W. Bruno, T. Leitner, Unequal Evolutionary Rates in the Human Immunodeficiency Virus Type 1 (HIV-1) Pandemic: the Evolutionary Rate of HIV-1 Slows Down When the Epidemic Rate Increases. *Journal of Virology*. **81**, 10625–10635 (2007).

2. J. Jorba, R. Campagnoli, L. De, O. Kew, Calibration of Multiple Poliovirus Molecular Clocks Covering an Extended Evolutionary Range. *J Virol*. **82**, 4429–4440 (2008).

3. D. Rejmanek, P. R. Hosseini, J. A. K. Mazet, P. Daszak, T. Goldstein, Evolutionary Dynamics and Global Diversity of Influenza A Virus. *Journal of Virology*. **89**, 10993–11001 (2015).

4. M. F. Boni, P. Lemey, X. Jiang, T. T.-Y. Lam, B. W. Perry, T. A. Castoe, A. Rambaut, D. L. Robertson, Evolutionary origins of the SARS-CoV-2 sarbecovirus lineage responsible for the COVID-19 pandemic. *Nature Microbiology*. **5**, 1408–1417 (2020).

5. L. van Dorp, M. Acman, D. Richard, L. P. Shaw, C. E. Ford, L. Ormond, C. J. Owen, J. Pang, C. C. S. Tan, F. A. T. Boshier, A. T. Ortiz, F. Balloux, Emergence of genomic diversity and recurrent mutations in SARS-CoV-2. *Infection, Genetics and Evolution*. **83**, 104351 (2020).

6. Y. Fujii, Y. H. Doan, Y. Suzuki, T. Nakagomi, O. Nakagomi, K. Katayama, Study of Complete Genome Sequences of Rotavirus A Epidemics and Evolution in Japan in 2012–2014. *Front. Microbiol.* **10** (2019), doi:10.3389/fmicb.2019.00038.

7. M. Yuan, T. Lu, C. Li, L. Lu, The Evolutionary Rates of HCV Estimated with Subtype 1a and 1b Sequences over the ORF Length and in Different Genomic Regions. *PLOS ONE*. **8**, e64698 (2013).
