# Supplementary material for: Risk of rapid evolutionary escape from biomedical interventions targeting SARS-CoV-2 spike protein
Source: PLoS One. 2021 Apr 28;16(4):e0250780. doi: 10.1371/journal.pone.0250780 (PMC8081162; doi:10.1371/journal.pone.0250780)
Supplement: S2 Table — (PDF) [file pone.0250780.s005.pdf]

Table S2. Antibody escape mutants with highest ACE2 binding affinities.

Antibody	% Change in Binding Affinity for Optimal Escape Mutant
S309	120%
EY6A	155%
S2A4	120%
S304	155%
S2M11	117%
B38	200%
C105	178%
P2B-2F6	141%
CB6	151%
CC12.1	200%
CC12.3	200%
CV30	200%
Fab 2-4	178%
COVA2-04	200%
COVA2-39	200%
S2H13	200%
S2H14	200%
S2E12	123%
REGN10933	178%
REGN10987	200%
