# Supplementary material for: Risk of rapid evolutionary escape from biomedical interventions targeting SARS-CoV-2 spike protein
Source: PLoS One. 2021 Apr 28;16(4):e0250780. doi: 10.1371/journal.pone.0250780 (PMC8081162; doi:10.1371/journal.pone.0250780)
Supplement: S3 Table — (PDF) [file pone.0250780.s006.pdf]

Table S3. Antibody structures used.

Antibody Name	Structure Type	Source
S309	Cryo-electron Microscopy and Crystal Structure	(*1*)
EY6A	Cryo-electron Microscopy and Crystal Structure	(*2*)
S2A4	Cryo-electron Microscopy and Crystal Structure	(*3*)
S304	Cryo-electron Microscopy and Crystal Structure	(*3*)
S2M11	Cryo-electron Microscopy	(*4*)
B38	Crystal Structure	(*5*)
C105	Cryo-electron Microscopy of complex. Crystal structure of antibody.	(*6*)
P2B-2F6	Crystal Structure	(*7*)
CB6	Crystal Structure	(*8*)
CC12.1	Crystal Structure	(*9*)
CC12.3	Crystal Structure	(*9*)
CV30	Crystal Structure	(*10*)
Fab 2-4	Cryo-electron Microscopy	(*11*)
COVA2-04	Crystal Structure	(*12*)
COVA2-39	Crystal Structure	(*12*)
S2H13	Cryo-electron Microscopy	(*3*)
S2H14	Cryo-electron Microscopy and Crystal Structure	(*3*)
S2E12	Cryo-electron Microscopy of complex. Crystal structure of antibody.	(*4*)

1. D. Pinto, Y.-J. Park, M. Beltramello, A. C. Walls, M. A. Tortorici, S. Bianchi, S. Jaconi, K. Culap, F. Zatta, A. De Marco, A. Peter, B. Guarino, R. Spreafico, E. Cameroni, J. B. Case, R. E. Chen, C. Havenar-Daughton, G. Snell, A. Telenti, H. W. Virgin, A. Lanzavecchia, M. S. Diamond, K. Fink, D. Veesler, D. Corti, Cross-neutralization of SARS-CoV-2 by a human monoclonal SARS-CoV antibody. *Nature*. **583**, 290–295 (2020).

2. D. Zhou, H. M. E. Duyvesteyn, C.-P. Chen, C.-G. Huang, T.-H. Chen, S.-R. Shih, Y.-C. Lin, C.-Y. Cheng, S.-H. Cheng, Y.-C. Huang, T.-Y. Lin, C. Ma, J. Huo, L. Carrique, T. Malinauskas, R. R. Ruza, P. N. M. Shah, T. K. Tan, P. Rijal, R. F. Donat, K. Godwin, K. R. Buttigieg, J. A. Tree, J. Radecke, N. G. Paterson, P. Supasa, J. Mongkolsapaya, G. R. Screaton, M. W. Carroll, J. Gilbert-Jaramillo, M. L. Knight, W. James, R. J. Owens, J. H. Naismith, A. R. Townsend, E. E. Fry, Y. Zhao, J. Ren, D. I. Stuart, K.-Y. A. Huang, Structural basis for the neutralization of SARS-CoV-2 by an antibody from a convalescent patient. *Nature Structural & Molecular Biology*. **27**, 950–958 (2020).

3. L. Piccoli, Y.-J. Park, M. A. Tortorici, N. Czudnochowski, A. C. Walls, M. Beltramello, C. Silacci-Fregni, D. Pinto, L. E. Rosen, J. E. Bowen, O. J. Acton, S. Jaconi, B. Guarino, A.

Minola, F. Zatta, N. Sprugasci, J. Bassi, A. Peter, A. D. Marco, J. C. Nix, F. Mele, S. Jovic, B. F. Rodriguez, S. V. Gupta, F. Jin, G. Piumatti, G. L. Presti, A. F. Pellanda, M. Biggiogero, M. Tarkowski, M. S. Pizzuto, E. Cameroni, C. Havenar-Daughton, M. Smithey, D. Hong, V. Lepori, E. Albanese, A. Ceschi, E. Bernasconi, L. Elzi, P. Ferrari, C. Garzoni, A. Riva, G. Snell, F. Sallusto, K. Fink, H. W. Virgin, A. Lanzavecchia, D. Corti, D. Veesler, Mapping Neutralizing and Immunodominant Sites on the SARS-CoV-2 Spike Receptor-Binding Domain by Structure-Guided High-Resolution Serology. *Cell*. **183**, 1024-1042.e21 (2020).

4. M. A. Tortorici, M. Beltramello, F. A. Lempp, D. Pinto, H. V. Dang, L. E. Rosen, M. McCallum, J. Bowen, A. Minola, S. Jaconi, F. Zatta, A. D. Marco, B. Guarino, S. Bianchi, E. J. Lauron, H. Tucker, J. Zhou, A. Peter, C. Havenar-Daughton, J. A. Wojcechowskyj, J. B. Case, R. E. Chen, H. Kaiser, M. Montiel-Ruiz, M. Meury, N. Czudnochowski, R. Spreafico, J. Dillen, C. Ng, N. Sprugasci, K. Culap, F. Benigni, R. Abdelnabi, S.-Y. C. Foo, M. A. Schmid, E. Cameroni, A. Riva, A. Gabrieli, M. Galli, M. S. Pizzuto, J. Neyts, M. S. Diamond, H. W. Virgin, G. Snell, D. Corti, K. Fink, D. Veesler, Ultrapotent human antibodies protect against SARS-CoV-2 challenge via multiple mechanisms. *Science* (2020), doi:10.1126/science.abe3354.

5. Y. Wu, F. Wang, C. Shen, W. Peng, D. Li, C. Zhao, Z. Li, S. Li, Y. Bi, Y. Yang, Y. Gong, H. Xiao, Z. Fan, S. Tan, G. Wu, W. Tan, X. Lu, C. Fan, Q. Wang, Y. Liu, C. Zhang, J. Qi, G. F. Gao, F. Gao, L. Liu, A noncompeting pair of human neutralizing antibodies block COVID-19 virus binding to its receptor ACE2. *Science*. **368**, 1274–1278 (2020).

6. C. O. Barnes, A. P. West, K. E. Huey-Tubman, M. A. G. Hoffmann, N. G. Sharaf, P. R. Hoffman, N. Koranda, H. B. Gristick, C. Gaebler, F. Muecksch, J. C. C. Lorenzi, S. Finkin, T. Hägglöf, A. Hurley, K. G. Millard, Y. Weisblum, F. Schmidt, T. Hatziioannou, P. D. Bieniasz, M. Caskey, D. F. Robbiani, M. C. Nussenzweig, P. J. Bjorkman, Structures of Human Antibodies Bound to SARS-CoV-2 Spike Reveal Common Epitopes and Recurrent Features of Antibodies. *Cell*. **182**, 828-842.e16 (2020).

7. B. Ju, Q. Zhang, J. Ge, R. Wang, J. Sun, X. Ge, J. Yu, S. Shan, B. Zhou, S. Song, X. Tang, J. Yu, J. Lan, J. Yuan, H. Wang, J. Zhao, S. Zhang, Y. Wang, X. Shi, L. Liu, J. Zhao, X. Wang, Z. Zhang, L. Zhang, Human neutralizing antibodies elicited by SARS-CoV-2 infection. *Nature*. **584**, 115–119 (2020).

8. R. Shi, C. Shan, X. Duan, Z. Chen, P. Liu, J. Song, T. Song, X. Bi, C. Han, L. Wu, G. Gao, X. Hu, Y. Zhang, Z. Tong, W. Huang, W. J. Liu, G. Wu, B. Zhang, L. Wang, J. Qi, H. Feng, F.-S. Wang, Q. Wang, G. F. Gao, Z. Yuan, J. Yan, A human neutralizing antibody targets the receptor-binding site of SARS-CoV-2. *Nature*. **584**, 120–124 (2020).

9. M. Yuan, H. Liu, N. C. Wu, C.-C. D. Lee, X. Zhu, F. Zhao, D. Huang, W. Yu, Y. Hua, H. Tien, T. F. Rogers, E. Landais, D. Sok, J. G. Jardine, D. R. Burton, I. A. Wilson, Structural basis of a shared antibody response to SARS-CoV-2. *Science*. **369**, 1119–1123 (2020).

10. N. K. Hurlburt, E. Seydoux, Y.-H. Wan, V. V. Edara, A. B. Stuart, J. Feng, M. S. Suthar, A. T. McGuire, L. Stamatatos, M. Pancera, Structural basis for potent neutralization of SARS-CoV-2 and role of antibody affinity maturation. *Nature Communications*. **11**, 5413 (2020).

11. L. Liu, P. Wang, M. S. Nair, J. Yu, M. Rapp, Q. Wang, Y. Luo, J. F.-W. Chan, V. Sahi, A. Figueroa, X. V. Guo, G. Cerutti, J. Bimela, J. Gorman, T. Zhou, Z. Chen, K.-Y. Yuen, P. D. Kwong, J. G. Sodroski, M. T. Yin, Z. Sheng, Y. Huang, L. Shapiro, D. D. Ho, Potent neutralizing antibodies against multiple epitopes on SARS-CoV-2 spike. *Nature*. **584**, 450–456 (2020).

12. N. C. Wu, M. Yuan, H. Liu, C.-C. D. Lee, X. Zhu, S. Bangaru, J. L. Torres, T. G. Caniels, P. J. M. Brouwer, M. J. van Gils, R. W. Sanders, A. B. Ward, I. A. Wilson, An Alternative Binding Mode of IGHV3-53 Antibodies to the SARS-CoV-2 Receptor Binding Domain. *Cell Reports*. **33**, 108274 (2020).
